# Genomic insights into a tripartite ancestry in the Southern Ryukyu Islands

**DOI:** 10.1017/ehs.2023.18

**Published:** 2023-07-10

**Authors:** Niall P. Cooke, Valeria Mattiangeli, Lara M. Cassidy, Kenji Okazaki, Kenji Kasai, Daniel G. Bradley, Takashi Gakuhari, Shigeki Nakagome

**Affiliations:** 1 School of Medicine, Trinity College Dublin, Dublin, Ireland; 2 Smurfit Institute of Genetics, Trinity College Dublin, Dublin, Ireland; 3 Department of Anatomy, Faculty of Medicine, Tottori University, Japan; 4 Toyama Prefectural Center for Archaeological Operations, Toyama, Japan; 5 Institute for the Study of Ancient Civilizations and Cultural Resources, College of Human and Social Sciences, Kanazawa University, Kanazawa, Japan

**Keywords:** Ancient genomics, Japanese archipelago, prehistoric to historic transition, gene flow, Nagabaka

## Abstract

A tripartite structure for the genetic origin of Japanese populations states that present-day populations are descended from three main ancestors: (1) the indigenous Jomon hunter–gatherers; (2) a Northeast Asian component that arrived during the agrarian Yayoi period; and (3) a major influx of East Asian ancestry in the imperial Kofun period. However, the genetic heterogeneity observed in different regions of the Japanese archipelago highlights the need to assess the applicability and suitability of this model. Here, we analyse historic genomes from the southern Ryukyu Islands, which have unique cultural and historical backgrounds compared with other parts of Japan. Our analysis supports the tripartite structure as the best fit in this region, with significantly higher estimated proportions of Jomon ancestry than mainland Japanese. Unlike the main islands, where each continental ancestor was directly brought by immigrants from the continent, those who already possessed the tripartite ancestor migrated to the southern Ryukyu Islands and admixed with the prehistoric people around the eleventh century AD, coinciding with the emergence of the Gusuku period. These results reaffirm the tripartite model in the southernmost extremes of the Japanese archipelago and show variability in how the structure emerged in diverse geographic regions.

## Graphical abstract

**Figure:**
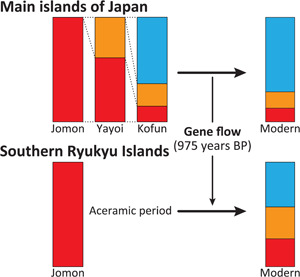

**Social media summary:** New genomic study sheds light on the emergence of a tripartite ancestral structure in the southern Ryukyu Islands.

## Introduction

Ancient genomic sequence data has enhanced and often changed our understanding of the origins of present-day populations throughout the world (Cassidy et al., 2020; Gamba et al., 2014; Haak et al., 2015). One such area is the Japanese archipelago, where analysis of pre- and proto-historic genomes has supported a tripartite model for the origin of present-day populations (Cooke et al., 2021). In this framework, present-day Japanese derive ancestry from three major sources: (1) the indigenous hunter–gatherer–fisher Jomon population, who lived across the archipelago for several millennia with little contact outside of the archipelago; (2) a Northeast Asian component observed in ancient individuals from the Amur River basin, northern China that arrived to the region with wet-rice farming during the Yayoi period (~3000 years ago); and (3) a major influx of ancestry resembling present-day East Asian populations (such as Han Chinese) that arrived during the Kofun period (~1700 years ago), which is associated with early imperial state formation.

While various models for the origins of Japanese populations have been previously proposed based on genetic, archaeological and linguistic evidence, including alternative versions of a tripartite structure (Chaubey & van Driem, 2020; van Driem, 2021), the ‘dual structure’ hypothesis, originally formulated based on craniofacial data (Hanihara, 1991), is the most widely known and enduring (Hudson et al., 2020). In this model, all Japanese populations are the descendants of the gradual mixing of two major sources of ancestry: the initial Jomon and subsequent Northeast Asian migrants during the Yayoi period. The dual structure model states that morphological similarities between the Ainu people from Hokkaido and the southernmost Ryukyu Islanders are due to their being descendents of the Jomon people with little to no ancestry from the later continental source population (Hanihara, 1991). Genetic heterogeneity is also observable between populations from the mainland of Japan and these two geographically distinct areas (Japanese Archipelago Human Population Genetics Consortium et al., 2012; Matsunami et al., 2021; Sakaue et al., 2020; Sato et al., 2014; Yamaguchi-Kabata et al., 2008). Furthermore, Jomon individuals have a higher genetic affinity to present-day Ainu, as well as Ryukyu Islanders, than those who are from the other parts of the archipelago (Gakuhari et al., 2020; Kanzawa-Kiriyama et al., 2019). Still, these observations can only explain variation in Jomon ancestry across Japan, rather than origins of continental ancestry.

The tripartite model proposed based on ancient genomic data has been shown to be a significantly better fit for the genetic ancestry of present-day Japanese individuals included in the Simon Genome Diversity Project (SGDP) panel when compared with the dual structure model (Cooke et al., 2021). However, the applicability of this framework in genetically distinct populations beyond the main islands has not yet been tested, as modern reference datasets are currently limited to a small subset of mainland Japanese that does not reflect the true heterogeneity of the region (1000 Genomes Project Consortium, 2015; GenomeAsia100K Consortium, 2019; Mallick et al., 2016). Assessing how this three-way admixture model varies in diverse parts of the archipelago could indicate differences in population origins and recent history within the Japanese archipelago.

The recent publication of sequence data from four historic individuals dating to ~150 years before present from the Nagabaka site on Miyako Island, Okinawa Prefecture (Robbeets et al., 2021) has made it possible to explore the ancestral profile of a recent population living in the southern Ryukyu Islands. The region has been recognised for its exceptional insular geography, history and culture, as highlighted by previous studies (Asato, 1991, 2003; Yamagiwa, 2022). It possesses distinct characteristics that set it apart from the main islands of Japan and even the northern parts of the Ryukyu Islands. One notable aspect is the prolonged duration of its prehistoric period, which lasted well into the eleventh century CE, when a unique regional culture known as Gusuku emerged. This extended period of insular and cultural isolation can be attributed to the absence of Yayoi, Kofun and other historic cultures that were widespread throughout most of the archipelago (Yamagiwa, 2022). As a result, the genomic data from the southern Ryukyu Islands presents a valuable opportunity to investigate the applicability and variability of the tripartite model and its formation process in the context of the origin of Japanese. In this study, we reexamine the tripartite structure using historical population data from the Ryukyu Islands and compare its ancestral profile with that of mainland Japanese populations.

## Results

**Figure 1.**
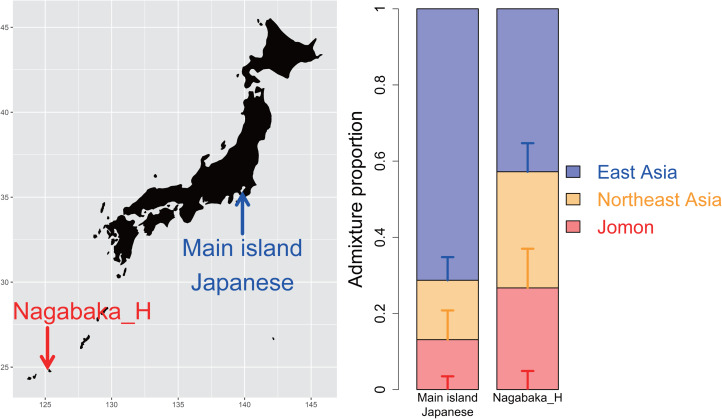
Tripartite structure in the southern Ryukyu Islands as determined by *qpAdm*. The bar graphs show the compositions of three distinct ancestors: Jomon (red), Northeast Asian (orange) and East Asian ancestry (blue). This model fits Nagabaka_H, a population of four historic individuals from the Nagabaka site, Miyako Island, southern Ryukyu Islands, with different proportions between the main islands and southern Ryukyu Islands.

Four historic individuals (NAG007, NAG035, NAG036 and NAG039; see Table S1) were recently sampled from skeletal remains excavated from the rock shelter and shell midden Nagabaka site on the northern peninsula of Miyako-jima Island, part of the Miyako Islands, Okinawa Prefecture (Figure 1; Robbeets et al., 2021). We first conducted a pairwise outgroup *f*_3_ analysis to establish their genetic affinity to other ancient and present-day Japanese samples. This analysis clearly defines clusters of Jomon, Yayoi and others including the Kofun, historic and present-day Japanese samples; within the third cluster, the historic individuals are further separated from the other samples owing to a higher genetic affinity to Jomon individuals than those from the Kofun and modern populations (Figure S1). We subsequently grouped these individuals together as a single population of ‘Nagabaka_H’, and using *qpAdm* further demonstrated that none of the ancient Japanese populations or present-day Japanese could be successfully modelled as a single source of ancestry (Table S2), ruling out the proposed idea that these inhabitants were the direct descendents of the Jomon. These results suggest differences in the ancestral composition between the Nagabaka individuals with those from the main islands.

To assess the suitability of the tripartite structure for Nagabaka_H, we modelled their genetic makeup by three different ancestral components: 12 Jomon individuals (Cooke et al., 2021; Gakuhari et al., 2020; Kanzawa-Kiriyama et al., 2019; McColl et al., 2018), two ancient Chinese individuals with a high level of ancestry from the northern Amur River region (WLR_BA_o and HMMH_MN) to represent Northeast Asian (NEA) ancestry (Ning et al., 2020) and present-day Han Chinese from the SGDP panel to represent East Asian ancestry (Mallick et al., 2016). The tripartite model successfully fits Nagabaka_H (tail probability, *p* = 0.591), with a breakdown of 26.7 ± 4.9% Jomon, 30.5 ± 10.3% NEA and 42.8 ± 7.5% East Asia (Figure 1). Interestingly, the proportion of the Jomon ancestry is approximately double in this historic population compared with in the Kofun (13.1 ± 3.5%) or modern Japanese (15.0 ± 3.8%) (Cooke et al., 2021).

The success in modelling a tripartite structure with the Nagabaka_H population does not rule out the possibility that alternative models, such as the one proposed by the dual structure hypothesis, may better fit their genetic profile. To comprehensively assess the suitability of the three-way admixture model in Nagabaka_H, we also tested fittings of all possible two-way admixture scenarios within the tripartite structure (i.e. Jomon and NEA, Jomon and East Asia, and East Asia and NEA). Two of these models (Jomon and NEA; NEA and East Asian) were rejected outright (*p* < 0.05); however a dual structure of Jomon and East Asia was found to be sufficient (*p* = 0.061). To conclude which model is the best fit, we calculated the *p*-values for the nested models for each comparison between the tripartite and each of the two-way models tested (Table 1). We find that the tripartite model fits the data significantly better than all of the nested two-way models (nested-*p* values < 0.05), including the dual structure between Jomon and Han (nested-*p* value = 0.0039). This is clear evidence that the tripartite structure is the best fitting model to explain the origins of the historic Ryukyu Island population.

**Table 1.** Comparing the tripartite admixture model to individual two-way models in the southern Ryukyu Islands. The fitness of the tripartite structure for ‘Nagabaka_H’ is compared with every possible combination of two-way models of the three proposed ancestral sources (Jomon, Northeast Asian and East Asian ancestry). *P-*values lower than 0.05 show that the two-way model is significantly less likely to fit than the three-way model.

Left populations: Target: Nagabaka_H Sources: • Jomon • NEA (WLR_BA_o and HMMH_MN) • EAS (Han)	Degrees of freedom	Tail probability	*χ*^2^	*p*-Value for the nested model (two-way vs. three-way model)
Models				
Three-way (Jomon, NEA, and EAS)	5	5.91 × 10^−1^	3.718	—
Two-way (Jomon and NEA)	6	8.57 × 10^−6^	33.454	4.95 × 10^−8^
Two-way (Jomon and EAS)	6	6.09 × 10^−2^	12.047	3.90 × 10^−3^
Two-way (NEA and EAS)	6	2.73 × 10^−5^	30.831	1.92 × 10^−7^

**Figure 2.**
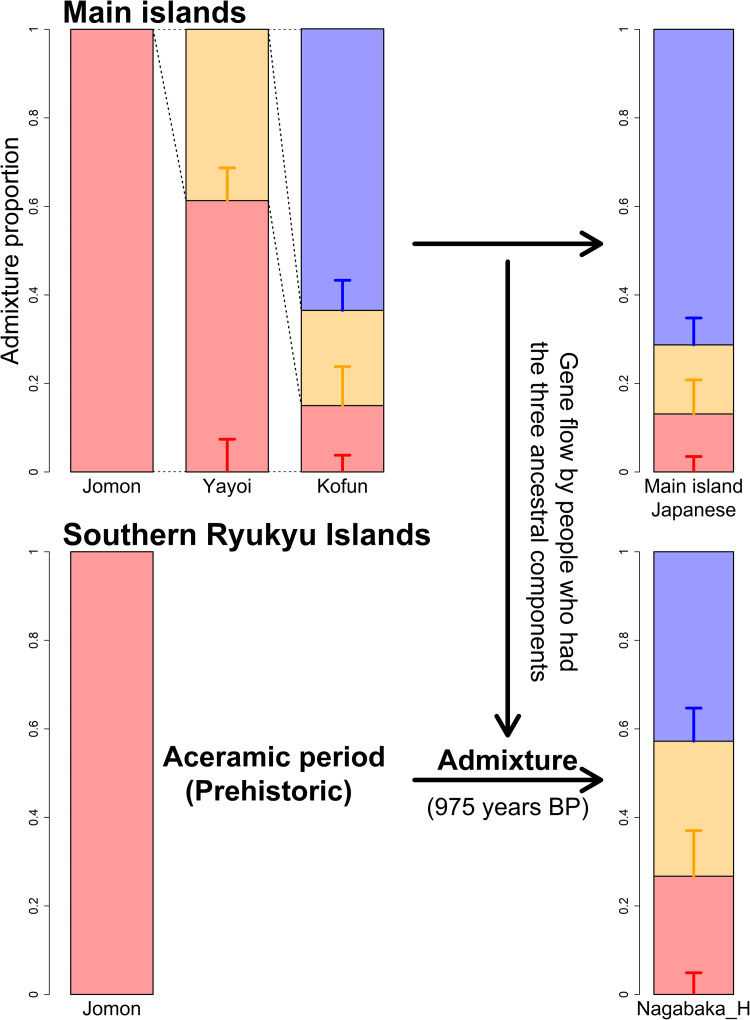
The difference in the formation process of the tripartite structure between the main islands and the southern Ryukyu Islands as estimated by *qpAdm*. A tripartite origin has been previously proposed for present-day main island Japanese (Cooke et al., 2021) in which ancestry is derived from the indigenous Jomon (red), a Northeast Asian component arriving in the Yayoi period (orange; as represented by two individuals, ‘WLR_BA_o’ and ‘HMMH_MN’ from (Ning et al., 2020) with ancestry from the Amur River Basin), and a later influx of East Asian ancestry (blue; as represented by present-day Han Chinese) during the Kofun period. The later non-Jomon continental sources of ancestry did not arrive in the southern Ryukyu Islands until the eleventh century AD, coinciding with the end of the prehistoric period and the beginning of the Gusuku period.

Considering the distinct historical and cultural backgrounds of the southern Ryukyu Islands (Yamagiwa, 2022), it is possible that the process of formation of the tripartite structure in this region differed from that of the main islands of Japan. Ancient genomic analysis has previously provided evidence that the prehistoric individuals in this region were genetically Jomon (Robbeets et al., 2021). Therefore, it is likely that the continental ancestry could have been brought by people who already possessed the two additional non-Jomon components. To test this hypothesis, we modelled Nagabaka_H by two-way admixture models between the Jomon and the Kofun or the modern Japanese individuals (Japanese in SGDP or JPT in 1000 Genome Phase 3; Table 2). All tested models fit the genetic ancestry of Nagabaka_H, with the admixture models being significantly better than the single ancestry models of either the Kofun or modern Japanese populations. These results suggest that additional Jomon ancestry is required to explain the genetic makeup of Nagabaka_H as shown in Figure 1. We further estimated that this admixture occurred 975 BP (or the eleventh century AD) using DATES (Figure S2), which is roughly consistent with the end of the Prehistoric (i.e. Aceramic) period and the beginning of the Gusuku period. Our analysis supports the idea that different regions have different histories in the formation of the tripartite structure (Figure 2), which in turn may have contributed to genomic variation across the Japanese archipelago.

**Table 2.** Fitting the two-way admixture models in the southern Ryukyu Islands. Jomon is fixed as the one source, while the other is represented by the Kofun or modern Japanese populations (Japanese in SDGP or JPT in 1000 Genome Phase 3). All three models show the tail probability larger than 0.05, supporting the admixture. The *p*-values for the nested models are calculated for the single ancestry model (i.e. no admixture) with each of Jomon or the additional source respectively.

Additional source	Jomon	Additional source	Tail probability	*p*-Value for the nested models	
				Jomon	Additional source
JpIw	0.197 ± 0.046	0.803 ± 0.046	0.386	0	3.69 × 10^−5^
Japanese (SGDP)	0.235 ± 0.048	0.765 ± 0.048	0.150	0	4.62 × 10^−6^
JPT (1000 Genome Phase 3)	0.225 ± 0.041	0.775 ± 0.041	0.365	0	4.23 × 10^−8^

## Discussion

Populations living within the Ryukyu Islands have been repeatedly shown to be genetically distinct from other groups living in the Japanese archipelago (Japanese Archipelago Human Population Genetics Consortium et al., 2012; Matsunami et al., 2021; Sakaue et al., 2020; Sato et al., 2014; Yamaguchi-Kabata et al., 2008). Nevertheless, we demonstrate through extensive admixture modelling that the tripartite structure for the origins of Japanese populations is successfully upheld in a population of historic individuals from the southern region. As was previously observed with representatives of populations from mainland Japan (Cooke et al., 2021), this model shows a significantly better fit than the long-standing ‘dual structure’ framework, which assumes that the Ryukuyan population is a direct descendent of the Jomon (Hanihara, 1991) (Table S2). This result suggests that both of the major genetic contributions made by post-Jomon migrants – first with Northeast Asian ancestry during the Yayoi period and subsequently with East Asian ancestry in the Kofun – were not limited to the main islands of Japan, but reached as far as the southernmost extremes of the archipelago. However, while these continental ancestries were previously shown to have arrived in mainland Japan at separate stages from distinct populations (Cooke et al., 2021), it appears that they were brought to the southern Ryukyu Islands at a much later stage by a single ancestral population who already possessed the tripartite structure and admixed with the prehistoric population (Figure 2).

An outgroup *f*_3_ analysis showed that the Nagabaka_H population shares a higher level of genetic drift with the Jomon than mainland individuals from the Kofun period or the present day (Figure S1). This population is subsequently shown to have a higher proportion of the Jomon ancestry of 26.7 ± 4.9% (Figure 1), which is approximately twice what is observed in the Kofun (13.1 ± 3.5%) or modern Japanese (15.0 ± 3.8%) (Cooke et al., 2021). Models of this population that incorporated additional Jomon ancestry were similarly found to be better fitting than those based solely on Kofun or modern Japanese ancestry. These results are consistent with the previous findings on a high genetic affinity between Jomon and present-day Ryukyuans (Gakuhari et al., 2020; Kanzawa-Kiriyama et al., 2019). Our study provides a more detailed picture of the admixture process that resulted in the excess of Jomon ancestry in the southern Ryukyu Islands.

Archeological records support an idea that the southern parts of Ryukyu Islands, including the Miyako Islands, underwent distinct changes compared with the main islands or even the northern Ryukyu Islands (Asato, 2003; Yamagiwa, 2022). While there are cultural connections indicated between the prehistoric Northern Ryukyus and the Jomon culture, these links are less evident in the southern Ryukyu Islands owing to the development of their own unique material culture (Yamagiwa, 2022). However, genetic analysis of the prehistoric individuals from the Nagabaka site (who are dated to 2600 and 3600 years BP; Robbeets et al., 2021), confirmed the presence of Jomon ancestry in this region.

Lifeways in the main islands of Japan began to undergo radical changes ~3000 years ago, initially from foraging to rice farming, and subsequently to state formation ~1700 years ago (Mizoguchi, 2013). In contrast, a completely different culture emerged in the southern Ryukyu Islands ~2500 years ago, known as Aceramic culture, which marked the final phase of the prehistoric period in the region (Asato, 1991; Yamagiwa, 2022). This culture was characterised by shell adzes and the absence of pottery production or utilisation, and it persisted for approximately 1700 years. Consequently, none of the cultural transitions that had occurred in the main islands of Japan since the Jomon period affected the southern Ryukyus until the eleventh century AD (Heinrich et al., 2015; Yamagiwa, 2022), when the Gusuku culture began and a large number of people migrated from the northern Ryukyus into the area (Matsunami et al., 2021; Sato et al., 2014). Although it remains unknown where the Aceramic culture came from, or whether or not the Jomon-like prehistoric people continued to inhabit the area during this cultural period (Yamagiwa, 2022), our estimate of when admixture between Jomon and main island Japanese ancestry occurred is consistent with the timing of this population movement (Figure S2). This result suggests that there was regional variation in the formation process of the tripartite structure across the Japanese archipelago.

This study is limited to a population of four individuals from Miyako Island in the southern Ryukyu Islands; however it is important to note that the Ryukyu Islands are not a genetically homogenous region (Sato et al., 2014), nor are the Miyako Islands themselves (Matsunami et al., 2021). Denser sampling in time and space is needed to see the true impact of migration on genetic profiles throughout the region. It would be of particular interest to assess what similarities or differences may exist in the tripartite breakdown between this population with other historic and present-day populations from throughout the archipelago when such data become available. This study once again shows the power of ancient genomics to change our understanding of population origins in different regions. It also shows the benefits of re-examining findings made and ideas proposed based on ancient genomics when new data relevant to the established conclusions become available.

## Materials and methods

### Data processing and preparation

BAM files for four historic individuals (NAG007, NAG035, NAG036 and NAG039) were downloaded from the European Nucleotide Archive and processed using the same bioinformatic pipeline as used in a previous study (Cooke et al., 2021). Analysis was conducted based on pseudo-haploid genotype data based on the SGDP (Mallick et al., 2016) autosomal transversions-only single nucleotide polymorphisms with a minor allele frequency of 1% and filtered for base quality of 30; this left 3,867,366 single nucleotide polymorphism sites used in analysis.

### F-Statistics

The *F*-statistics were calculated using *qp3Pop* (v300), part of the AdmixTools v6.0 package (Patterson et al., 2012). ‘Mbuti’ (*n* = 4) were used as an outgroup. Heatmaps for *f*_3_ values were created using the heatmap.2 package in R.

### Admixture modelling

Admixture events were modelled using *qpAdm* v1000 in the AdmixTools v6. 0 package (Haak et al., 2015; Patterson et al., 2012) using the parameter option of ‘allsnps: YES’. ‘Right’ populations included as outgroups in the analysis consisted of eight Eurasian populations: Sardinian (*n* = 3), Kusunda (*n* = 2), Papuan (*n* = 14), Dai (*n* = 4), Ami (*n* = 2), Naxi (*n* = 3), Tianyuan (*n* = 1) (Yang et al., 2017), Chokhopani (*n* = 1) (Jeong et al., 2016) and Mal'ta (*n* = 1) (Raghavan et al., 2014). Nested *p-*values were calculated in R using the formula: 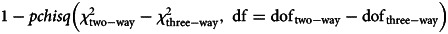, where 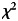 is the chi-square value under a given model, and dof is the degrees of freedom.

### Dates analysis

We used DATES v753 (Narasimhan et al., 2019) to estimate the time of the admixture event in the Nagabaka_H individuals. We used the Jomon individuals and the Japanese in Tokyo in the 1000 Genome phase 3 data (1000 Genomes Project Consortium, 2015) as the source of this admixture model. The estimated date in generation was converted into years with the assumption of 25 years per generation. The parameter settings that we used are as follows: binsize, 0.001; maxdis, 1.0; runmode, 1; mincount, 1; and lovalfit, 0.45. The standard error was estimated from a weighted block jackknife method.

## Research transparency and reproducibility

The data that support the findings of this study have been already published in previous studies (Cooke et al., 2021; Robbeets et al., 2021).
